# Leukocytapheresis in Chronic Myeloid Leukemia With Leukostasis

**DOI:** 10.7759/cureus.12375

**Published:** 2020-12-30

**Authors:** Babita Raghuwanshi, Sunil Chouhan, Anuj Jain

**Affiliations:** 1 Transfusion Medicine, All India Institute of Medical Sciences Bhopal, Bhopal, IND; 2 Physiology, All India Institute of Medical Sciences Bhopal, Bhopal, IND; 3 Anaesthesiology, All India Institute of Medical Sciences Bhopal, Bhopal, IND

**Keywords:** leukostasis, chronic leukemias, therapeutic leukocytapheresis

## Abstract

Leukostasis in acute and chronic leukemias leads to increased cell burden and increased blood viscosity. Therapeutic leukocytapheresis is an automated procedure aimed at white blood cell depletion, and it thereby reduces the complications associated with increased blood viscosity, such as thrombotic events and mortality. In this report, we present the case of a 25-year-old patient with leukostasis and splenic laceration who was treated with therapeutic leukocytapheresis with symptomatic relief in leukostasis.

## Introduction

Hematological malignancies can have diverse implications; the leukocyte count is one of the most important factors. Hyperleukocytosis is an emergent condition requiring therapy without delay. A high leukocyte count predisposes the patient to complications, mainly in two ways; firstly, arising from high viscosity and the resulting abnormality of microcirculation; secondly, from tumor lysis syndrome (TLS). The blast cells are less deformable, and therefore the increase in the number of blast cells leads to clogging and obstruction of microcirculation and an increase in blood viscosity, hypoxic endothelial injury, and cytokine release with pulmonary and neurological symptoms. Therapeutic leukocytapheresis is a grade-2B indication for leukemia with a white blood cell (WBC) count of >100 x 10^9^/L with leukostasis as per the American Society of Apheresis [1]. Therapeutic leukocytapheresis is performed to reduce the elevated WBC count to prevent leukostasis and hyperviscosity.

The intermittent flow cell separators use the principle of cell separation based on gradient density centrifugation. Therapeutic leukocytapheresis is an automated procedure aimed at white blood cell depletion in patients of leukemia with leukostasis.

## Case presentation

A 25-year-old woman, who had been recently diagnosed with chronic myeloid leukemia (CML) with BCR-ABL gene fusion and awaiting initiation of chemotherapy, presented with left subcostal tenderness following blunt trauma to the abdomen. Although pale, she was hemodynamically stable at presentation. A CT of the abdomen revealed a longitudinal splenic laceration, but no other organ injury was reported. Surgical consultation was obtained, and nonoperative management was suggested since the patient was hemodynamically stable. The patient was kept under strict surgical surveillance in the critical care unit for any clinical deterioration.

Blood investigations showed a leukocyte count of 450.11 x 10^3^/μL, neutrophil count of 385.64 x 10^3^, eosinophil of 22.43 x 10^3^, basophil of 22.84 x 10^3^, hemoglobin of 11.6 gm/dl, hematocrit of 33.8%, mean corpuscular volume of 82.1, and a platelet count of 425 x 10^3^ microliters; the blast count was found to be 6.3%.

The patient had a history of fatigue, malaise, tenderness in the left hypochondriac region, night sweats, weight loss (6-7 kg over a period of six months), loss of appetite, dyspnea on exertion (grade-2), headache, dizziness, and tinnitus. These symptoms had been gradually progressing over the past four to five months. She did not have a history of loss of consciousness or fever.

An interdepartmental discussion among transfusion medicine, anesthesiology, and surgery elicited a suggestion that there was an urgent need for cytoreduction therapy. Initiation of chemotherapy with such a high WBC count could have precipitated a TLS. Besides, the patient was also showing signs of leukostasis (dyspnea, dizziness). Novotny scoring suggested a high probability of leukostasis [2]. Taking into consideration the high total leukocyte count (TLC), leukostasis, and a potential need for emergency surgery due to splenic laceration, cytoreduction therapy by leukocytapheresis was decided upon. The tumor burden would be reduced by leukocytapheresis, which would lead to a consequent reduction in the chances of TLS along with improved blood viscosity and microcirculation. 

Informed consent was obtained from the patient for leukocytapheresis. Pre-procedure investigations were done; electrolytes (sodium: 138 mmol/L; potassium: 3.7 mmol/L; chloride: 105 mmol/L); prothrombin time (PT): 12.3/12.3; international normalised ratio (INR): 1.0; activated partial thromboplastin time (APTT): 30.6/30.2; liver and renal function tests were within normal limits.

Under strict aseptic precautions, a double-lumen central venous catheter was inserted into the right internal jugular vein under ultrasound guidance. As a standard protocol, 1.5 times the patient's blood volume was processed. Acid citrate dextrose (ACD) was used as an anticoagulant with a ratio of 1:14. Therapeutic leukocytapheresis was carried out on intermittent flow cell separator COBE Spectra (CaridianBCT, Lakewood, CO).

The apheresis kit was primed by using a whole blood unit as undiluted packed red blood cells (PRBC) had the risk of increasing blood viscosity. A calcium gluconate infusion was started by adding 10 ml of 10% calcium gluconate to every 100 ml of 0.9% NaCl at the rate of 10-15 ml/hr; serum calcium levels were monitored hourly. During the procedure, the patient's vitals were constantly monitored. Normal saline was used to maintain blood volume during the procedure. A total of 5,150 ml of blood was processed and 1,000 ml of the product was collected. After the procedure, the WBC count was reduced to 300.5 x 10^3^, thereby reducing the count by 150 x 10^3^ (a 34% reduction). Along with WBC count, the hemoglobin was also reduced to 9.3 gm/dl and platelet count decreased by 70 x 10^3^.

After leukocytapheresis, the patient showed improvements in symptoms of leukostasis (dyspnoea, headache, dizziness). The patient did not need to undergo any surgery. During her stay in the hospital, tyrosine kinase inhibitor (TKI) chemotherapy (imatinib) for the management of CML was initiated [3].

## Discussion

CML with hyperleukocytosis has important clinical implications and is usually managed with chemotherapy (CTX). The usual chemotherapeutic agents used are imatinib, dasatinib, and nilotinib [3]. However, initiation of chemotherapy alongside hyperleukocytosis may lead to TLS, which is a life-threatening situation. Besides, due to altered blood viscosity and obstruction of microcirculation, important organs like the lungs, brain, and liver are predisposed to dysfunction. The extent of alteration in physiology is directly proportional to the TLC. Leukocytapheresis along with hydration may help reduce the risk of TLS and complications associated with hyperviscosity, especially in patients with very high TLC [3]. Leukocytapheresis leads to rapid cytoreduction and does not affect the coagulation parameters [4].

Surgery in patients with myeloproliferative disorders has its challenges, which are compounded if the surgery is emergent since there is limited time to optimize the patient. CML patients have to undergo lifelong chemotherapy and are at risk of chemotherapy-associated complications. Often, the patients develop a blast crisis (blast count of more than 30%) and it is at this time that they are at maximum risk of developing TLS and hyperviscosity-related complications.

In CML, patients develop massive organomegaly and are at greater risk of solid organ injury, either spontaneous or due to blunt trauma to the abdomen. If the TLC is not raised much, the perioperative risk of complications is less. If the TLC is very high and surgery entails the handling of the abdominal viscera like the liver and spleen, the chances of TLS are high; if the patient is hemodynamically stable, leukocytapheresis may be especially useful. Usually, 1.5-2 blood volumes are processed [5]. A complete blood count (CBC) is done before beginning the procedure and upon completion of leukocytapheresis [6,7].

## Conclusions

The case under discussion had a high TLC and massive splenomegaly; fortunately, the splenic laceration did not make the patient hemodynamically unstable. The blood volume processed in leukocytapheresis is dependent on the initial WBC count and the patient's symptoms. By performing leukocytapheresis in this patient, we reduced the chances of TLC, which provided symptomatic relief to the patient from leukostasis and reduced the chances of other complications that could have resulted from surgery or chemotherapy initiation.
